# Evaluation of Peripheral Blood Circulation Disorder in Scleroderma Patients Using an Optical Sensor with a Pressurization Mechanism

**DOI:** 10.1371/journal.pone.0159611

**Published:** 2016-08-01

**Authors:** Yoshiki Yamakoshi, Sei-ichiro Motegi, Osamu Ishikawa

**Affiliations:** 1 Graduate School of Science and Technology, Gunma University, Tenjin, Kiryu, Gunma, Japan; 2 Graduate School of Medicine, Gunma University, Showa, Maebashi, Gunma, Japan; Singapore Immunology Network, SINGAPORE

## Abstract

Blood circulation function of peripheral blood vessels in skin dermis was evaluated employing an optical sensor with a pressurization mechanism using the blood outflow and reflow characteristics. The device contains a light source and an optical sensor. When applied to the skin surface, it first exerts the primary pressure (higher than the systolic blood pressure), causing an outflow of blood from the dermal peripheral blood vessels. After two heartbeats, the pressure is lowered (secondary pressure) and blood reflows into the peripheral blood vessels. Hemoglobin concentration, which changes during blood outflow and reflow, is derived from the received light intensity using the Beer–Lambert law. This method was evaluated in 26 healthy female volunteers and 26 female scleroderma patients. In order to evaluate the blood circulation function of the peripheral blood vessels of scleroderma patients, pressurization sequence which consists of primary pressure followed by secondary pressure was adopted. Blood reflow during the first heartbeat period after applying the secondary pressure of 40mmHg was (mean±SD) 0.059±0.05%mm for scleroderma patients and 0.173±0.104%mm for healthy volunteers. Blood reflow was significantly lower in scleroderma patients than in healthy volunteers (p<0.05). This result indicates that the information necessary for assessing blood circulation disorder of peripheral blood vessels in scleroderma patients is objectively obtained by the proposed method.

## Introduction

Systemic sclerosis (SSc), also known as scleroderma, is a disease characterized by fibrosis of skin and internal organs, vascular disorder, and immunological abnormalities[1–5]. Microvascular damage and dysfunction are the earliest morphological and functional markers of scleroderma. Fingers and toes are particularly prone to persistent digital ischemia caused by microcirculatory disorder[4,5]. In this disorder, even a minor injury can lead to a digital ulcer. When infected, such ulcers have a tendency to worsen and increase the size and depth. Thus, early appropriate treatment is important to control the condition. Therefore, reliable methods and devices to evaluate the status of microcirculation are required to prevent the development of digital ulcers and ensure their early, effective treatments.

In order to evaluate the microvascular functions, several methods have been developed. Laser Doppler method measures the blood flow velocity from the phase change of the reflected laser beam[6–8]. Optical coherence tomography, which is utilized for the visualization of tissue structure[9–11], is applied to the measurement of microvascular blood flow[12]. Ultrasound Doppler technique is used to visualize blood flow as a motion picture[13,14]. Blood flow and blood vessel structure of nailhold is visualized by nailhold capillaroscopy[15,16]. Thermography is also used in the evaluation and diagnosis of microcirculation disorder[17,18]. Pulse oximeter have been shown to be affected by fibrosis in their use in patients with SSc[19].

We have proposed an optical sensor with a pressurization mechanism for characterization of the blood circulation of dermal peripheral blood vessels in flow-mediated dilation[20]. This device consists of a LED and a photodetector, which are the same with reflection-type pulse oximeter[21,22]. However, the optical sensor is pressed by a static pressure to the skin surface using a moving-coil type actuator. When the applied pressure (primary pressure P1) is higher than the systolic pressure, the blood in dermal peripheral blood vessels, which consist of arteriole, capillary and venula, forced to flow out. Then, the pressure is decreased (secondary pressure P2), causing the blood to reflow into the peripheral blood vessels. The light scattered from the skin dermis is received by an optical sensor, and the change in hemoglobin concentration along the optical path during the pressurization of primary pressure and secondary pressure is estimated using the Beer–Lambert law. Indexes of outflow and reflow characteristic are evaluated. In this paper, an optical sensor with a pressurization mechanism was implemented to assess the blood circulation of dermal peripheral blood vessels in 26 scleroderma patients and 26 healthy female volunteers.

## Materials and Methods

### Measurement of hemoglobin concentration

**Fig 1:**
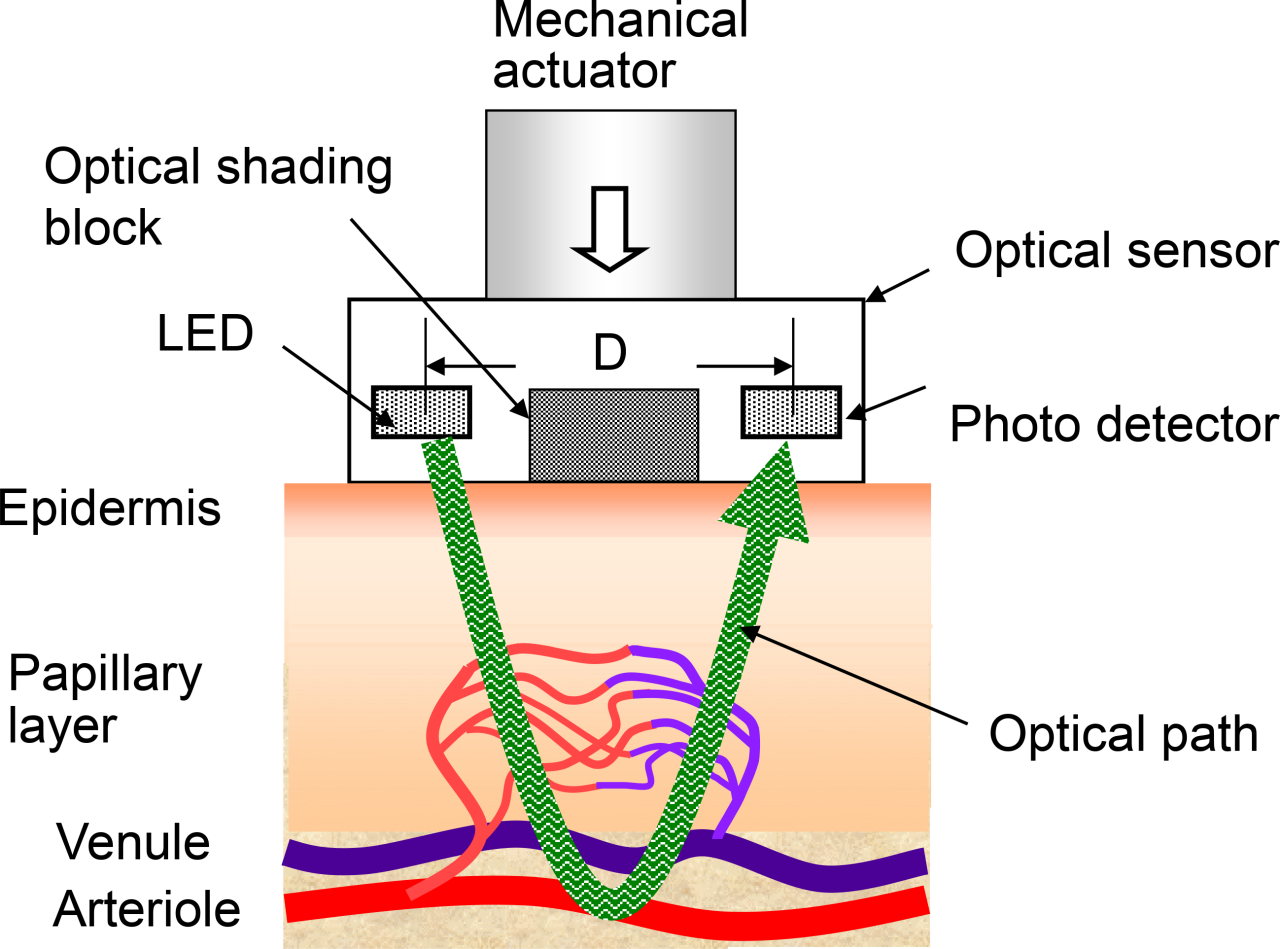
Schematic diagram of an optical sensor with a pressurization mechanism.

Fig 1 shows a schematic diagram of an optical sensor with a pressurization mechanism. An optical sensor contains a LED, a photodetector, and an optical shading block placed at the center of the optical sensor. Optical shading block is adopted so that both the direct light from the LED to the photodetector and the scattered light propagating close to the skin surface are blocked. Light emitted by the LED is scattered from the dermis and is received by a photodetector. The light intensity received by the photodetector is

$$I(t)=KA_{T}I_{i}\exp(-(\varepsilon_{o}C_{o}+\varepsilon_{d}C_{d})l),$$ (1)

where *A*_*T*_ and *I*_*i*_ are the light absorption of the tissue and the light intensity of the LED, respectively. *ε*_*0*_, *ε*_*d*_, *C*_*0*_, and *C*_*d*_ are the attenuation coefficient of oxyhemoglobin, attenuation coefficient of deoxyhemoglobin, concentration of oxyhemoglobin, and concentration of deoxyhemoglobin, respectively. *l* is the optical path length and *K* is a constant.

If we assume that the attenuation coefficient of oxyhemoglobin is equal to that of deoxyhemoglobin by selecting the wavelength of light, Eq (1) may be rewritten as follows:

$$I(t)=KA_{T}I_{i}\exp(-\epsilon Cl),$$ (2)

where *ε* is the attenuation coefficient of oxyhemoglobin and deoxyhemoglobin. *C* is the sum of oxyhemoglobin concentration and deoxyhemoglobin concentration.

If pressurization of the optical sensor continues long enough so that the whole blood flows out from the dermal peripheral blood vessels, the received light intensity can be calculated as follows:

$$I_{0}=KA_{T}I_{i}.$$ (3)

From Eqs (2) and (3), a parameter of hemoglobin concentration, *C*_*Hb*_, defined by the multiplication of *C* and *l*, is

$$C_{Hb}=Cl=-\frac{1}{\varepsilon }{log}_{e}\left(\frac{I(t)}{I_{0}}\right).$$ (4)

The change in hemoglobin concentration (i.e., the change of blood) in the dermal peripheral blood vessels caused by pressurization is estimated from the change in the received light intensity.

### Measurement apparatus

**Fig 2:**
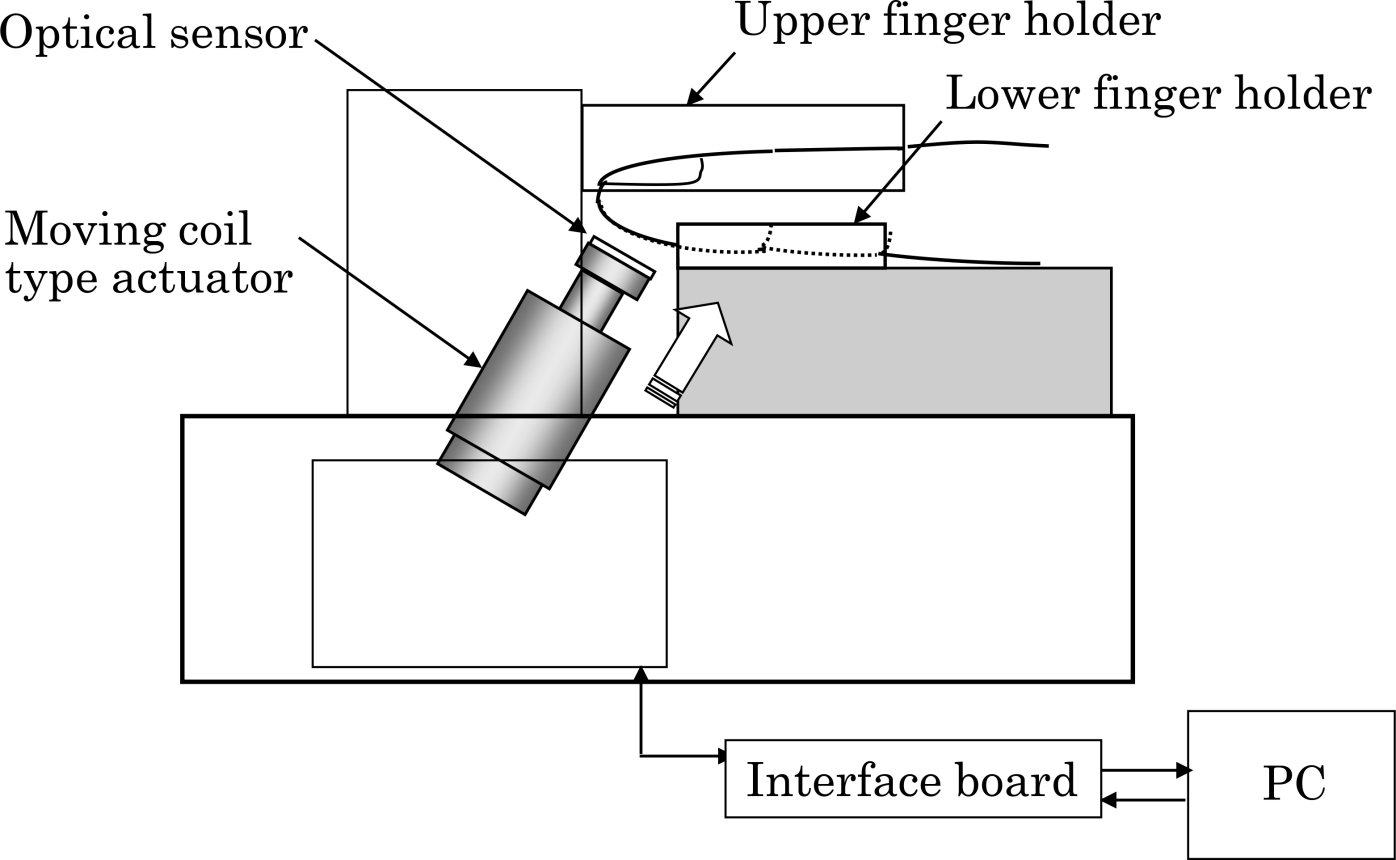
Cross-sectional image of the optical sensor with a pressurization mechanism.

**Fig 3:**
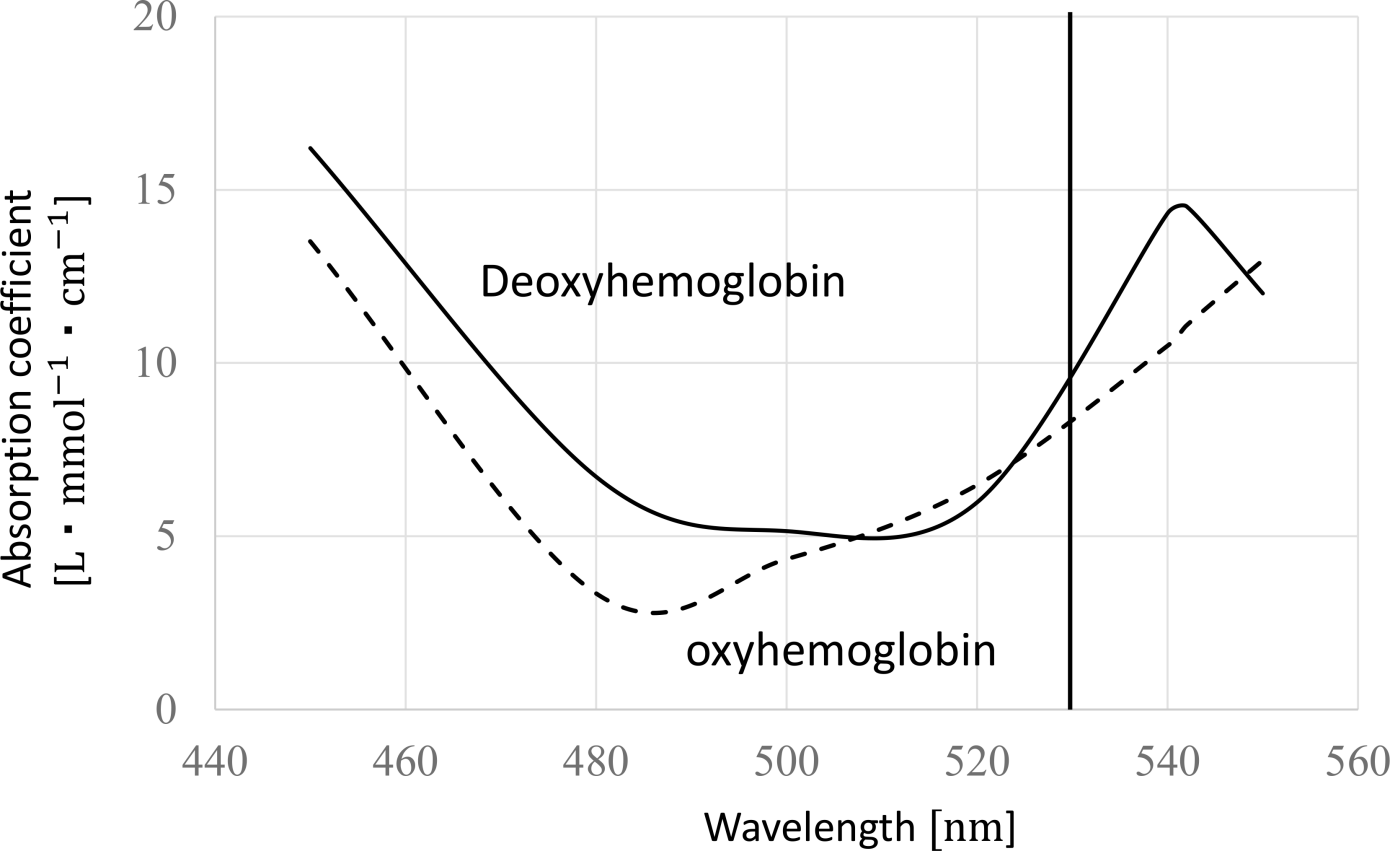
Photo absorption coefficient of oxyhemoglobin and deoxyhemoglobin.

Fig 2 shows a cross-sectional image of the optical sensor with a pressurization mechanism, constructed for the examination of the blood circulation of fingertip peripheral blood vessels. A right-hand finger is held between the upper and bottom finger holder. The optical sensor is pressed by a moving coil-type actuator and the force of the actuator and the timing of pressurization are controlled by a PC. Emitted LED light is chopped with a frequency of 1kHz; the received light intensity is detected by a lock-in amplifier in order to increase the signal-to-noise ratio of the signal. The wavelength of the LED is 530nm with half wavelength of 40nm so that oxyhemoglobin and deoxyhemoglobin show almost the same attenuation coefficient. Fig 3 shows attenuation coefficient of oxyhemoglobin and deoxyhemoglobin for the wavelength of 450–550 nm [23]. Oxygen saturation of capillary blood which is measured for newborns is 80.5% (Mean value)[24]. The light attenuation coefficients of oxyhemoglobin and that of deoxyhemoglobin are 9.7 and 8.7[L*mol^-1^*cm^-1^] respectively. Hence, the light absorption of blood with the oxygen saturation of 80.5% (capillary blood) is 9.45[L*mol^-1^*cm^-1^]. The difference from the light absorption of oxyhemoglobin which corresponds to that of artery blood is 2.6%. This difference shows that total hemoglobin concentration is estimated with high accuracy by using the light of 530nm wavelength. Because the measurement is finished within several minutes, it is assumed that the oxygen saturation of blood is a constant value during the experiment. Therefore, the small difference of attenuation coefficients between oxyhemoglobin and deoxyhemoglobin can be ignored in the measurement of hemoglobin concentration change caused by pressurization.

**Fig 4:**
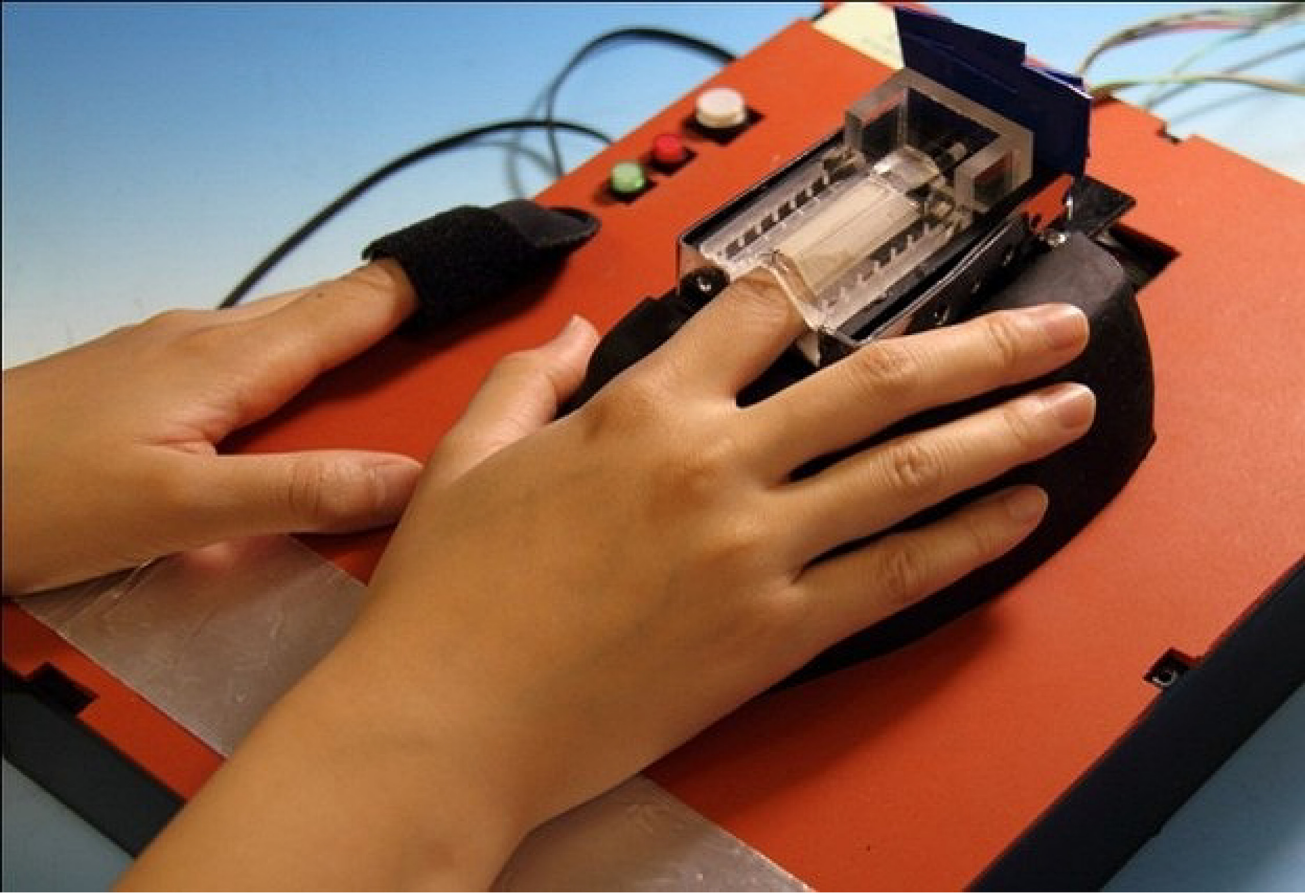
Photograph of the sensing system.

Fig 4 shows a photograph of the sensing system. The right forefinger is held between the lower and upper finger holders, and the blood outflow and reflow characteristics are measured by the optical sensor. A reflection-type optical sensor is also attached to the left forefinger and the change in blood volume caused by the heartbeat is monitored. Pressurizations with the primary pressure and secondary pressure start in synchronization with the heartbeat signal in order to suppress the effect of the blood pressure of the arterioles. Heart beat signal is monitored ad is counted automatically by an optical sensor which is attached to the left forearm.

### Time chart of pressurization

**Fig 5:**
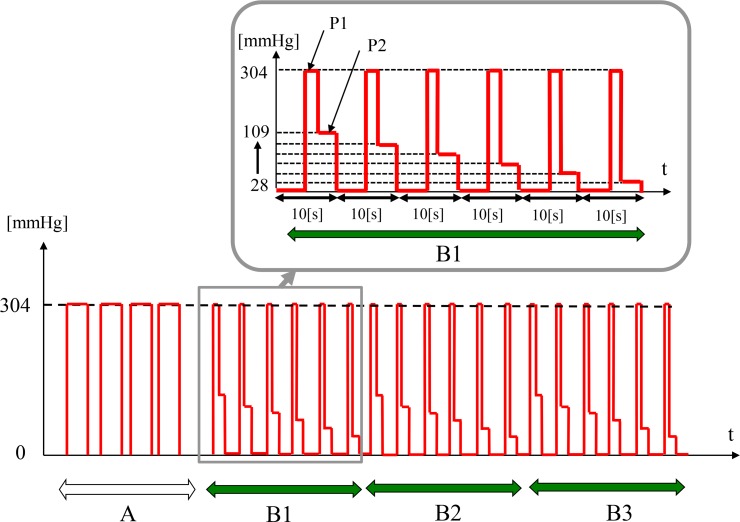
Time chart of pressure applied to the skin surface using the optical sensor with a pressurization mechanism. In section A, the absorption of light by the dermal tissue is estimated. In section B1-B3, blood reflow characteristics of the peripheral blood vessels are recorded by changing P2.

Fig 5 shows a time chart of the pressure applied to the skin surface using the optical sensor with a pressurization mechanism. This sequence consists of the following two steps.

#### Step1: Measurement of the light absorption of tissue

A primary pressure P1, which was much higher than the systolic pressure, was applied for 10s to cause the outflow of blood from the peripheral blood vessels. The absorption of light by the dermis was estimated from the blood outflow curve. The primary pressure P1 was set to 304mmHg in this experiment. This measurement was repeated 4 times and the light absorbed by the tissue was obtained as an averaged value (Section A in Fig 5).

#### Step2: Measurement of hemoglobin concentration under pressurization

A sequence of pressurizations (the primary pressure followed by the secondary pressure) was applied. In this sequence, the primary pressure (304mmHg) was applied during two heartbeat periods to make a part of the blood flow out from the dermal peripheral blood vessels. By this pressurization, hemoglobin concentration usually decreases to less than 30% of the initial value, which is small enough to monitor the blood reflow caused by the pressurization of secondary pressure. Blood reflow characteristics of the peripheral blood vessels were obtained using the secondary pressure P2 at six different levels: 28, 40, 53, 64, 88, and 109mmHg (Section B1 in Fig 5). These secondary pressures having approximately equal pressure difference were chosen, because dominant blood reflow observed in this pressure range for healthy volunteers. Blood outflow and blood reflow measurements were repeated every 10s (including a short break) as shown in Fig 5. The same measurement was repeated three times (B1-B3). Minimum value of the secondary pressure is 28mmHg in this experimental apparatus because the actuator to apply pressure does not work well due to the friction of the mechanism.

**Fig 6:**
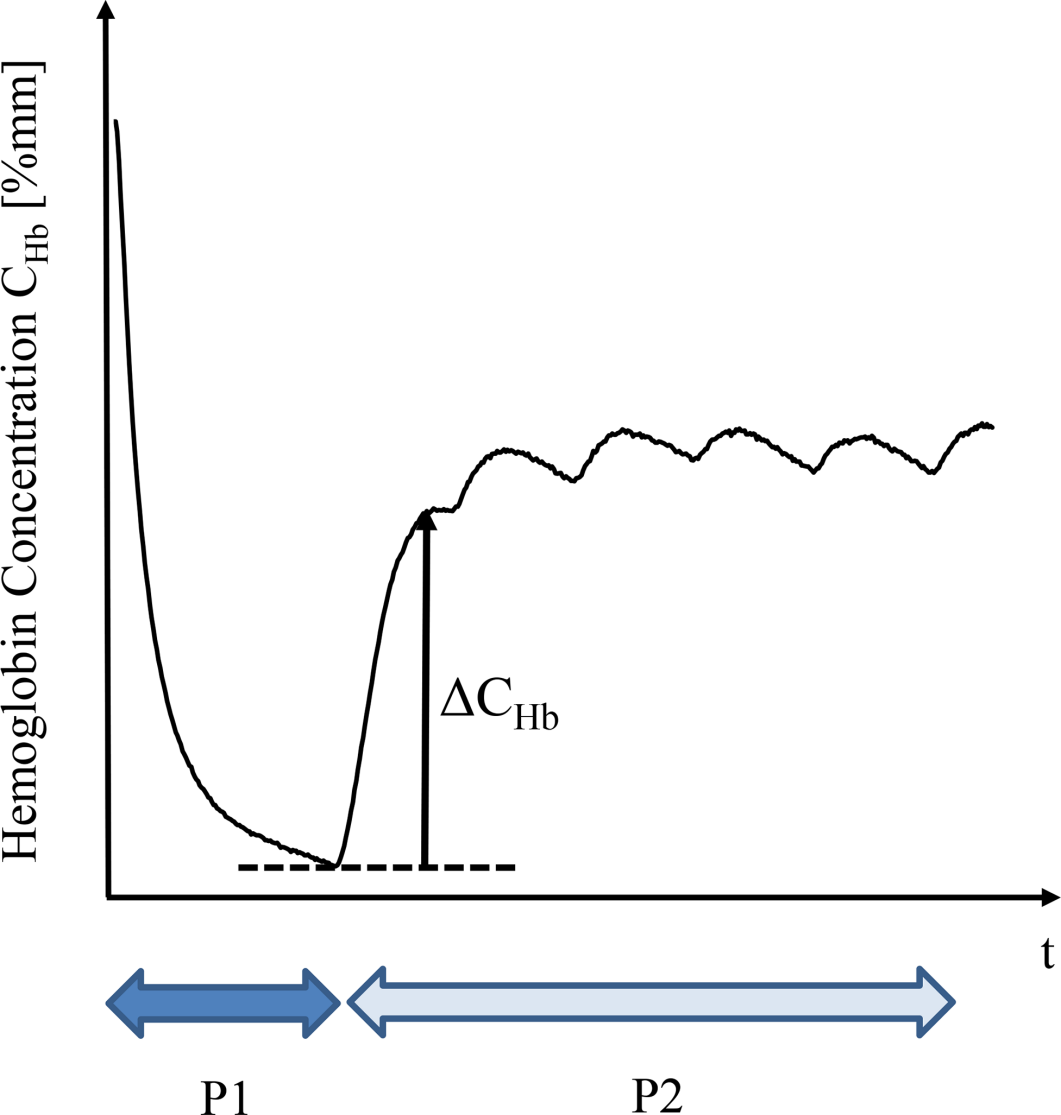
An index of blood reflow characteristic *ΔC*_*Hb*_.

Typical curve of hemoglobin concentration, when the pressures P1 and P2 are applied, is shown in Fig 6. Blood flows out by the pressure P1, and starts to re-flow when the pressure is set to P2. In order to evaluate the blood reflow characteristic, an index of blood reflow characteristic *ΔC*_*Hb*,_ is introduced. *ΔC*_*Hb*_ is defined as the recovery in hemoglobin concentration during the first heartbeat period of secondary pressure, as is shown in Fig 6. Large *ΔC*_*Hb*_ shows sufficient blood reflow under the secondary pressure.

## Subjects

26 female healthy volunteers [age (mean±SD): 69.7±4.13 years old] and 26 female scleroderma patients (59.9±13.9 years old) were measured. All patients fulfilled the criteria of scleroderma proposed by the American College of Rheumatology (1980)[25] and the American College of Rheumatology/European League against Rheumatism Classification Criteria (2013)[26]. All our scleroderma patients suffered peripheral circulation disorders, such as Raynaud’s phenomenon, but did not have digital ulcers. This study was approved by the Institutional Review Board of Gunma University. All healthy individuals and scleroderma patients provided written informed consent before participation.

## Results

**Fig 7:**
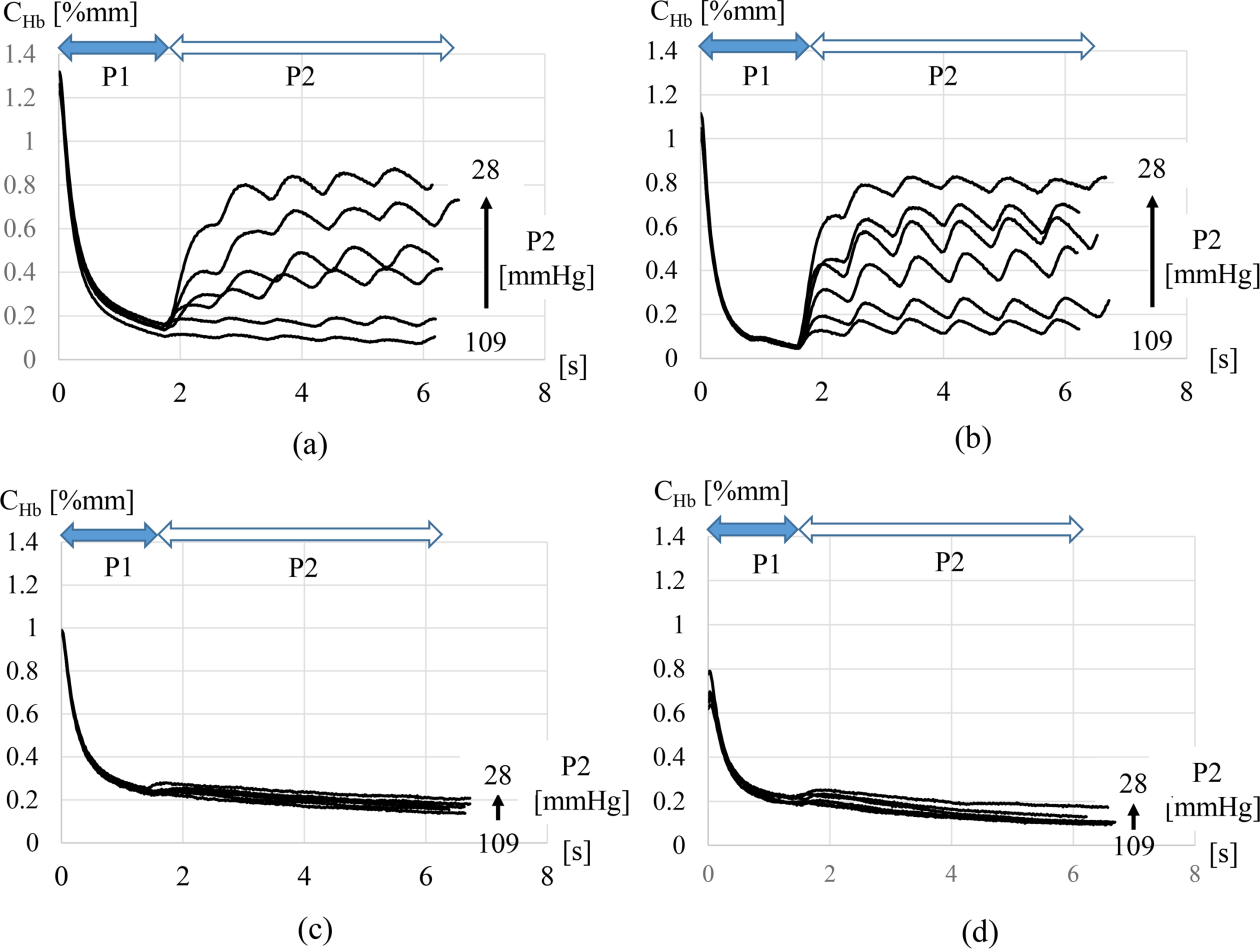
Hemoglobin concentration *C*_*Hb*_ measured using the proposed method. Figs (a) and (b) are *C*_*Hb*_ which are observed for two healthy volunteers. Figs (c) and (d) are *C*_*Hb*_ which were observed in two scleroderma patients.

Fig 7 shows representative results of the hemoglobin concentration *C*_*Hb*_ measured using the proposed method. Fig 7A and 7B present *C*_*Hb*_ in two healthy volunteers. Fig 7C and 7D are *C*_*Hb*_ in two scleroderma patients. The horizontal axis shows the time and the vertical axis shows *C*_*Hb*_. When the primary pressure P1 was applied for two heartbeat periods, blood flowed out from the peripheral blood vessels. This blood outflow was shown as a rapid decrease in hemoglobin concentration *C*_*Hb*_. Then, the secondary pressure P2 was applied to cause the blood to reflow into the peripheral blood vessels. In the healthy volunteers, *C*_*Hb*_ increased significantly with a decrease in the secondary pressure; the inherent periodical fluctuations caused by the heartbeat were observed (Fig 7A and 7B). However, neither a significant blood reflow nor a clear heartbeat signal was observed during the secondary pressure period of scleroderma patients (Fig 7C and 7D). Moreover, the concentration of hemoglobin in these patients decreased with time even when the secondary pressure was set to the minimum secondary pressure 28mmHg. Hemoglobin concentration at the initial time (t = 0) shows hemoglobin concentration which is recovered after some rest time returning the pressure to zero. Clear difference of reflow characteristic under the secondary pressure, which was observed between two groups, was not shown in the hemoglobin concentration at t = 0, though the hemoglobin concentration of scleroderma patients is lower than that of healthy volunteers. This result clearly showed that pressurization sequence was effective in order to detect the difference of blood reflow characteristic between scleroderma patients and healthy volunteers.

**Fig 8:**
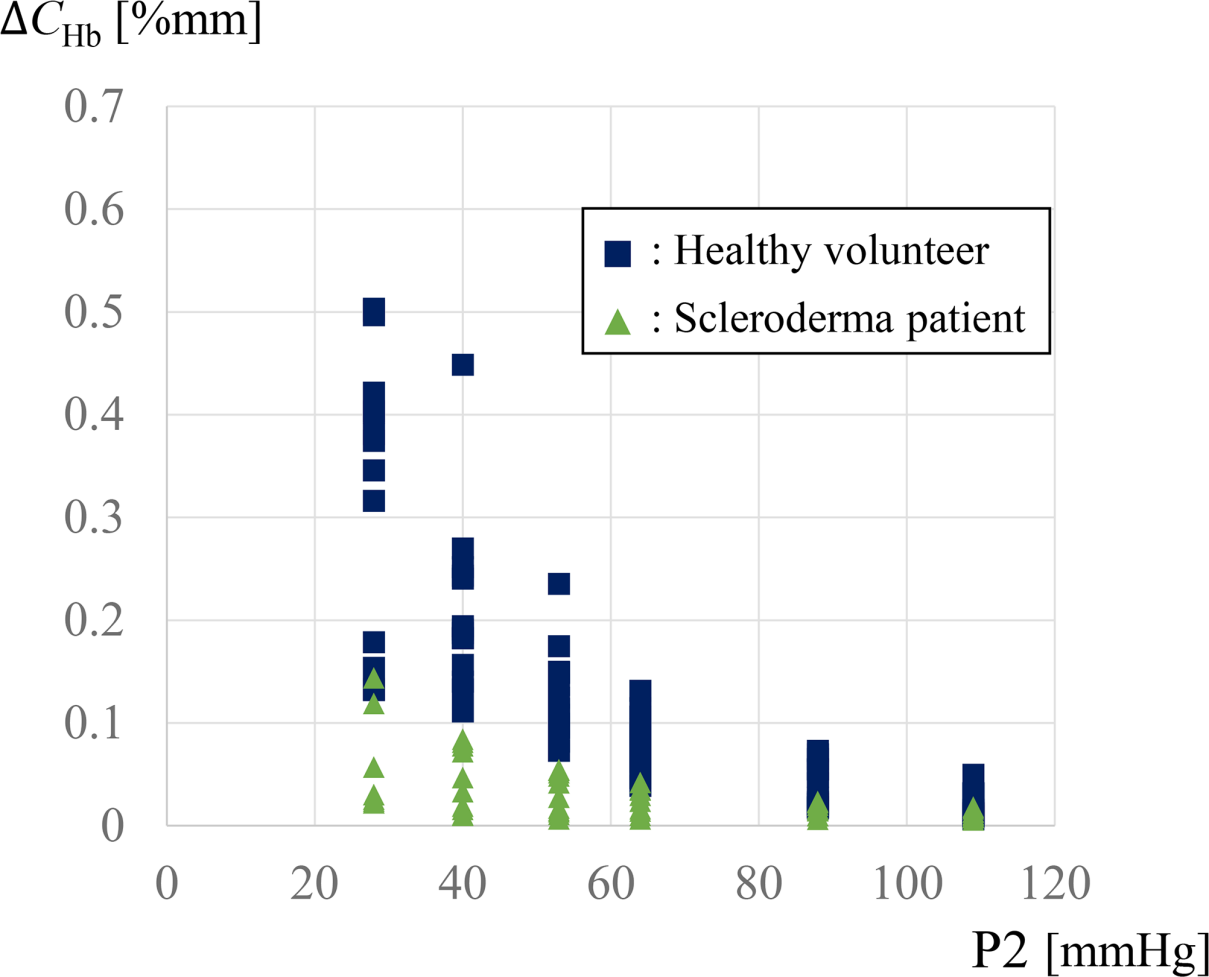
*ΔC*_*Hb*_ measured in four healthy volunteers and four scleroderma patients.

Fig 8 shows *ΔC*_*Hb*_ obtained from four healthy volunteers and four scleroderma patients. The horizontal axis shows the secondary pressure and the vertical axis shows *ΔC*_*Hb*_. Four successive measurement data are shown. A clear recovery of hemoglobin concentration was observed when the secondary pressure was decreased for healthy volunteers. However, the recovery of hemoglobin concentration under the secondary pressure was much lower than that in healthy volunteers, although *ΔC*_*Hb*_ increased slightly with a decrease in the secondary pressure.

**Fig 9:**
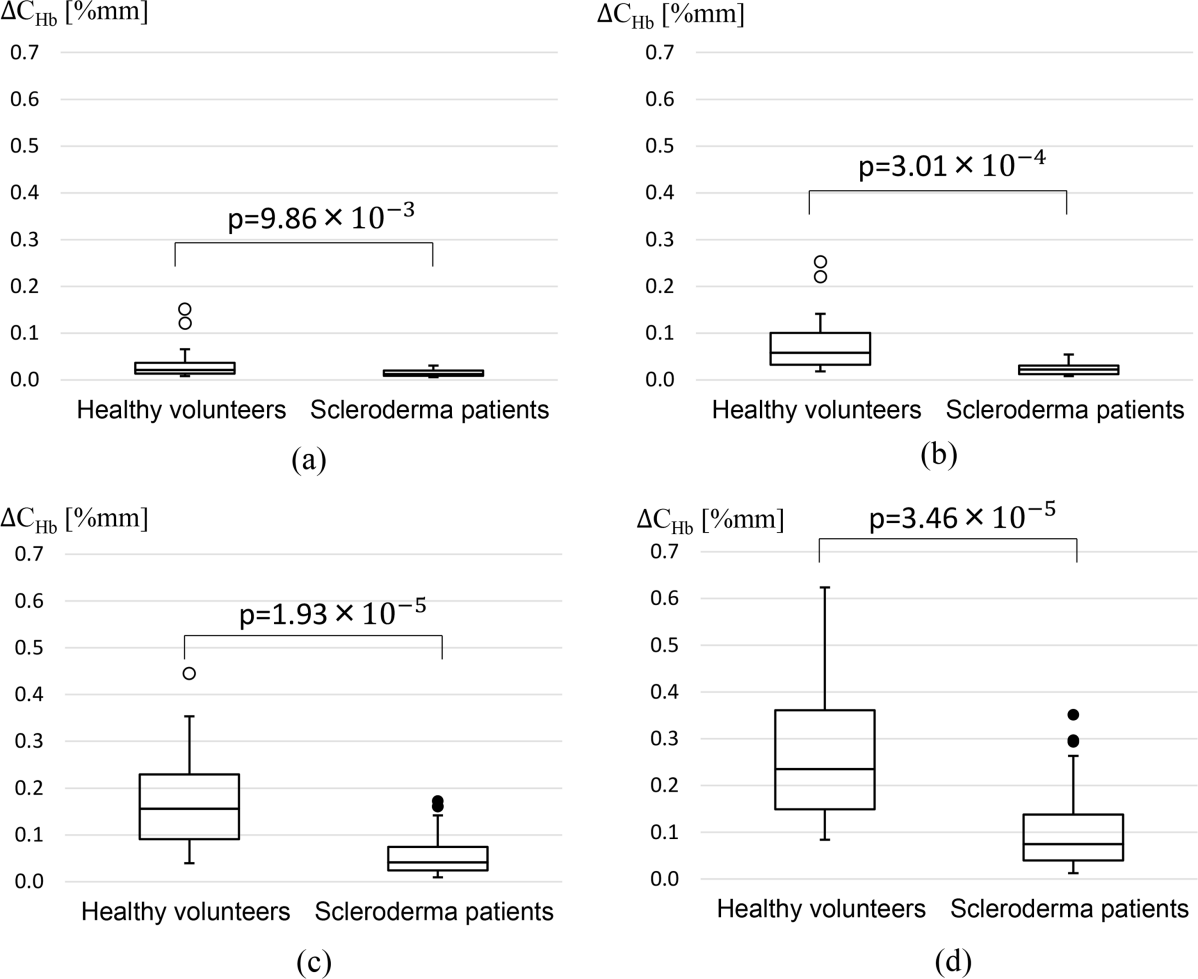
*ΔC*_*Hb*_ measured in 26 healthy volunteers and 26 scleroderma patients. (a):P2 = 88mmHg, (b):P2 = 64mmHg, (c):P2 = 40mmHg, and (d):P2 = 28mmHg.

Fig 9 shows *ΔC*_*Hb*_ measured in 26 healthy volunteers and 26 scleroderma patients. Results obtained for P2 of 88, 64, 40 and 28mmHg are shown in Fig 9A–9D, respectively. Statistical analysis between two groups was performed by Student's t test. Although a significant difference was observed in four secondary pressures, p value was the smallest when the secondary pressure was set at 40mmHg (Fig 9C). *ΔC*_*Hb*_ for the secondary pressure 40mmHg was (mean±SD) 0.059±0.05%mm for scleroderma patients and 0.173±0.104%mm for healthy volunteers. *ΔC*_*Hb*_ measured in 26 healthy volunteers and 26 scleroderma patients is also shown in S1 Dataset. These results demonstrate that blood circulation disorder of peripheral blood vessels in scleroderma patients can be detected by the proposed method.

**Fig 10:**
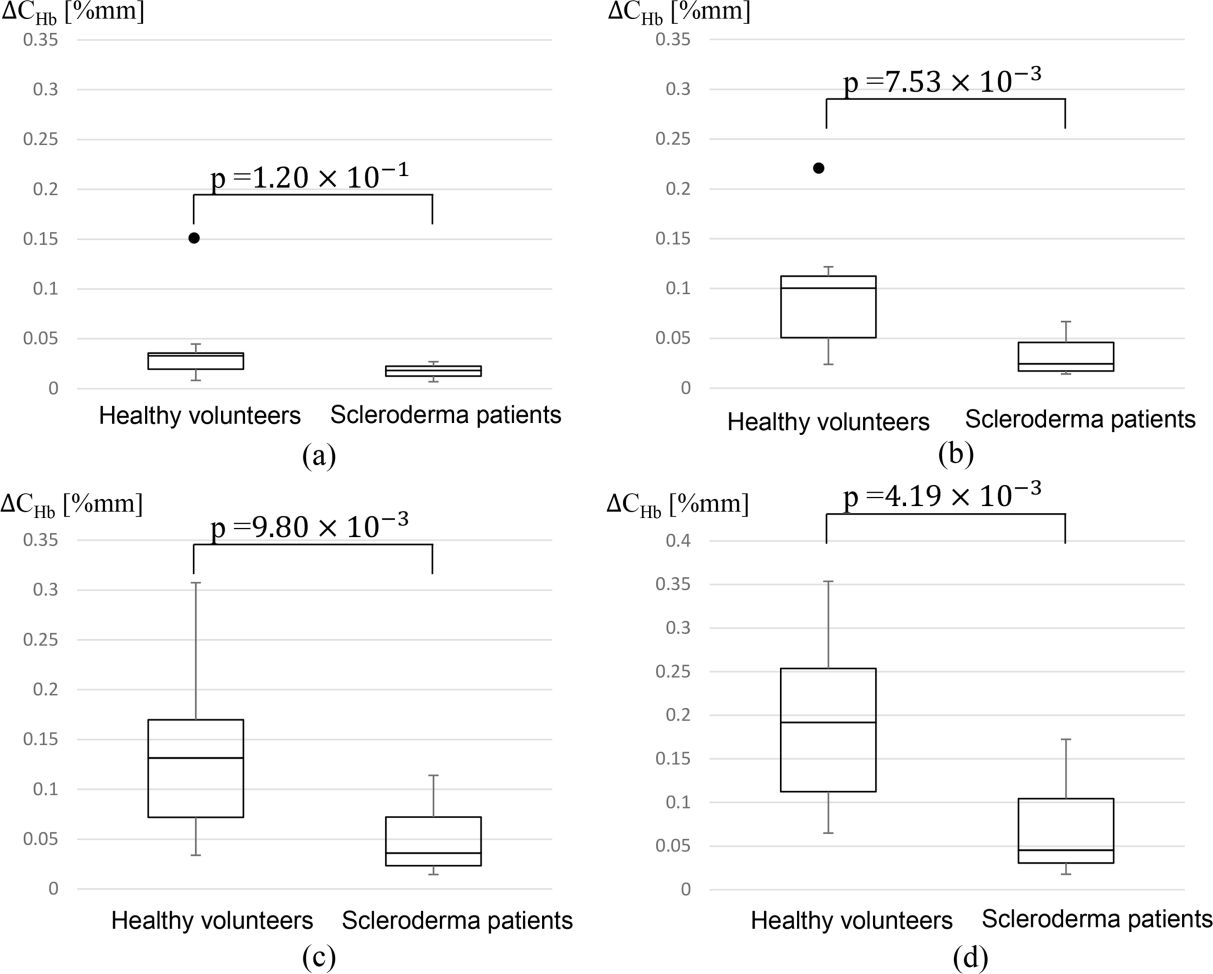
*ΔC*_*Hb*_ measured in healthy volunteers aged 65 to 75 years old (N = 10) and scleroderma patients aged 65 to 75 years old (N = 10). (a):P2 = 88mmHg, (b):P2 = 64mmHg, (c):P2 = 40mmHg, and (d):P2 = 28mmHg.

Age distribution of healthy volunteers is different from that of scleroderma patients. Hence, ten healthy volunteers who aged 65 to 75 years old and ten scleroderma patients of the same age range were compared. Fig 10 shows the results. Fig 10A–10D are the results for P2the secondary pressure of 88, 64, 40 and 28mmHg, respectively. There is no significance difference when P2 is set to 88mmHg, however, clear difference was seen between two groups for P2 = 64, 40 and 28mmHg.

## Discussion

We propose a novel method to evaluate the blood circulation disorder of peripheral blood vessels in scleroderma patients. The method employs a reflection-type optical sensor in combination with pressure to the skin. At first, blood outflows after applying the primary pressure P1 (higher than the systolic pressure). Then, the pressure is lowered to the secondary pressure P2 to cause the blood to reflow into the peripheral blood vessels. The changes in hemoglobin concentration during the procedure are calculated from the received light intensity using the Beer–Lambert law. The method is noninvasive; the outflow and reflow characteristics of peripheral blood vessels are obtained without any discomfort to the patients. The procedure takes only a few minutes and the measuring equipment is easy to use.

The blood circulation of peripheral blood vessels is also affected by the skin temperature. Hence, we monitored the skin temperature (using an infrared thermometer) before and after the experiment. Subjects whose skin temperature was below 30°C were asked to keep their hands in water at 40°C for 1 min to recover the blood flow. The skin temperature of four healthy volunteers and four scleroderma patients was elevated to >30°C using this method. Blood pressure was also measured using an electromanometer. However, the correlation coefficient between Δ*C*_*Hb*_ and systolic blood pressure was 0.012. This result showed that the blood reflow characteristics were independent of the systolic blood pressure.

In scleroderma patients with microcirculatory disturbance, neither a significant blood reflow nor a clear heartbeat signal was observed during the secondary pressure period. Moreover, the amount of blood reflow during the first heartbeat period of the secondary pressure was significantly lower in scleroderma patients than in healthy individuals. These results suggested that an abnormal blood reflow in the fingertip peripheral blood vessels was associated with blood circulation disturbances in scleroderma patients. A t-test was performed between scleroderma patients and heathy volunteers *ΔC*_*Hb*_ for different secondary pressure P2. P value was the smallest when P2 was set at 40mmHg, although a significant difference was also obtained for 88, 64 and 28mmHg.

The peripheral microvascular disorder is a dynamic event in scleroderma; it is an efficient predictor of disease development and progression[27]. The vascular changes associated with scleroderma are classified into two groups: destructive vasculopathy and proliferative/obliterative vasculopathy[28]. Destructive vasculopathy affects small vessels and manifests itself early in the course of scleroderma as progressive loss of capillaries and insufficient angiogenesis. We have previously reported that atherosclerotic changes are not primarily involved in the development of digital ulcers [4]. These data, in conjunction with our results demonstrating the suppressed blood reflow in scleroderma patients, suggest a progressive loss of finger capillaries in scleroderma. Our results also suggest that *ΔC*_*Hb*_ is a useful marker for the evaluation of the severity of the blood circulation disorder of peripheral blood vessels in scleroderma patients. Based on our results, we believe that this measurement technique can be applied for the detection, diagnosis and evaluation of peripheral vascular dysfunctions in many diseases, such as SSc, peripheral arterial disease and thromboangiitis obliterans. In addition, in clinically, we suggest that this measurement technique might use for the assessment of the efficacy of the drugs for the treatment with peripheral vascular dysfunction.

Feature of the proposed method is that the blood flow and reflow characteristics of peripheral blood circulation under applying pressurization can be monitored quantitatively, which cannot be achieved by other methods. Since blood vessel in peripheral blood circulation is difficult to detect by optical methods due to its size of blood vessel. The Doppler method which is widely adopted to monitor blood flow cannot be applied because blood flow direction is not fixed in the region of interest. In the proposed method, hemoglobin concentration in peripheral blood circulation is monitored by introducing a pressurization mechanism to a reflection-type optical measurement method. Change of blood amount along the optical path when pressure is applied from skin surface is measured by Beer-Lambert law. The proposed method is the only method that can evaluate peripheral circulation blood flow and reflow characteristics.

## Conclusions

Blood reflow characteristics under a static pressure were evaluated using an optical sensor with a pressurization mechanism. Two different types of pressure were applied in sequence. The primary pressure P1 caused the outflow of blood from dermal peripheral blood vessels, whereas the secondary pressure P2 caused the blood reflow. The reflow of blood into the peripheral blood vessels in scleroderma patients was significantly lower than that in the healthy volunteers. This result shows that the proposed method can provide valuable data for assessing the blood circulation disorder of peripheral blood vessels in scleroderma patients.

## Supporting Information

S1 Dataset *ΔC*_*Hb*_ measured in 26 healthy volunteers and 26 scleroderma patients. P2 are 88, 64, 53, 40, and 28mmHg.
(PDF)

## References

[1] Jinnin M. Mechanisms of skin fibrosis in systemic sclerosis. J Dermatol 2010;37(1):11–25. 10.1111/j.1346-8138.2009.00738.x 20175837

[2] Asano Y. Future treatments in systemic sclerosis. J Dermatol 2010;37(1):54–70. 10.1111/j.1346-8138.2009.00758.x 20175840

[3] Toki S, Motegi S, Yamada K, Uchiyama A, Ishikawa O. Demographic and clinical features of autoimmune thyroid disorder in Japanese patients with systemic sclerosis. J Dermatol 2014;41(12):1053–1057. 10.1111/1346-8138.12698 25387898

[4] Motegi S, Toki S, Hattori T, Yamada K, Uchiyama A, Ishikawa O. No association of atherosclerosis with digital ulcers in Japanese patients with systemic sclerosis: Evaluation of carotid intima-media thickness and plaque characteristics. J Dermatol 2014;41(7):604–608. 10.1111/1346-8138.12532 24942495

[5] Motegi S, Toki S, Yamada K, Uchiyama A, Ishikawa O. Elevated plasma homocysteine level is possibly associated with skin sclerosis in a series of Japanese patients with systemic sclerosis. J Dermatol 2014;41(11):986–991. 10.1111/1346-8138.12642 25293445

[6] Wardell K, Jakobsson A, Nilsson GE. Laser-Doppler perfusion imaging by dynamic light-scattering. IEEE Trans Biomed Eng 1993;40(4):309–319. 8375866 10.1109/10.222322

[7] Briers J. Laser Doppler, speckle and related techniques for blood perfusion mapping and imaging. Physiol Meas 2001;22(4):R35–R66. 11761081 10.1088/0967-3334/22/4/201

[8] Rosato E, Borghese F, Pisarri S, Salsano F. Laser Doppler perfusion imaging is useful in the study of Raynaud's phenomenon and improves the capillaroscopic diagnosis. J Rheumatol. 2009 10;36(10):2257–63. 10.3899/jrheum.090187 19684154

[9] Chen Z, Milner T, Srinivas S, Wang X, Malekafzali A, vanGemert M, et al Noninvasive imaging of in vivo blood flow velocity using optical Doppler tomography. Opt Lett 1997;22(14):1119–1121. 18185770 10.1364/ol.22.001119

[10] Wang R, An L, Francis P, Wilson D. Depth-resolved imaging of capillary networks in retina and choroid using ultrahigh sensitive optical microangiography. Opt Lett 2010;35(9):1467–1469. 10.1364/OL.35.001467 20436605 PMC2864924

[11] Ring HC, Mogensen M, Hussain AA, Steadman N, Banzhaf C, Themstrup L, et al Imaging of collagen deposition disorders using optical coherence tomography. J Eur Acad Dermatol Venereol. 2015 5;29(5):890–8. 10.1111/jdv.12708 25178655

[12] Chen Z, Milner T, Srinivas S, Wang X, Malekafzali A, van Gemert M, et al Nelson J. Noninvasive imaging of in vivo blood flow velocity using optical Doppler tomography. Opt Lett 1997;22(14):1119–1121. 18185770 10.1364/ol.22.001119

[13] Goertz D, Yu J, Kerbel R, Burns P, Foster F. High-frequency 3-D color-flow imaging of the microcirculation. Ultrasound Med Biol 2003;29(1):39–51. 12604116 10.1016/s0301-5629(02)00682-8

[14] Christopher D, Burns P, Starkoski B, Foster F. A high-frequency pulsed-wave doppler ultrasound system for the detection and imaging of blood flow in the microcirculation. Ultrasound Med Biol 1997;23(7):997–1015. 9330444 10.1016/s0301-5629(97)00076-8

[15] Cutolo M, Grassi W, Cerinic MM. Raynaud's Phenomenon and the Role of Capillaroscopy. Arthritis & Rheumatism 2003;48(11):3023–3030. 14613262 10.1002/art.11310

[16] Cutolo M. Sulli A, Secchi ME, Paolino S, Pizzorni C. Nailhold capillaroscopy is useful for the diagnosis and follow-up of autoimmune rheumatic diseases. A future tool for the analysis of microvascular heart involvement?. Rheumatology 2006;45(Suppl 4):v43–iv46. 10.1093/rheumatology/kel310 16980724

[17] Schlager O, Gschwandtner M, Herberg K, Frohner T, Schillinger M, Koppensteiner R, et al Correlation of infrared thermography and skin perfusion in Raynaud patients and in healthy controls. Microvasc Res 2010;80(1):54–57. 10.1016/j.mvr.2010.01.010 20144625

[18] Zhang HD, He Y, Wang X, Shao HW, Mu LZ, Zhang J. Dynamic infrared imaging for analysis of fingertip temperature after cold water stimulation and neurothermal modeling study. Comput Biol Med 2010;40(7):650–656. 10.1016/j.compbiomed.2010.05.003 20542263

[19] Akdogan A, Kilic L, Dogan I, Karadag O, Bilgen SA, Kiraz S, et al Effect of capillaroscopic patterns on the pulse oximetry measurements in systemic sclerosis patients. Microvasc Res. 2015 3;98:183–6. 10.1016/j.mvr.2014.02.002 24530379

[20] Yamakoshi Y, Kotani K, Taniguchi N, Miwa T. Characterization of skin dermis microcirculation in flow-mediated dilation using optical sensor with pressurization mechanism. Med Biol Eng Comput 2013;51(5):497–505. 10.1007/s11517-012-1017-2 23274949

[21] Sinex J. Pulse oximetry: principles and limitations. Am J Emerg Med 1999;17(1):59–66. 9928703 10.1016/s0735-6757(99)90019-0

[22] Lawson D, Norley I, Korbon G, Loeb R, Ellis J. Blood-flow limits and pulse oximeter signal-detection. Anesthesiology 1987;67(4):599–603. 3662092 10.1097/00000542-198710000-00032

[23] Zijlstra W, Buursma A, Meeuwsen-van der Roest W, Absorption Spectra of Human Fetal and Adult Oxyhemoglobin, De-Oxyhemoglobin, Carboxyhemoglobin, and Methemoglobin, Clin. Chem. 1991;37(9):1633–1638. 1716537

[24] Ješić M, Ješić M, Krstajić T, Vujnović Z, Čutura N, Maglajlić S, Reference Values of Capillary Blood Saturation in Neonates and its Difference from Pulse Oximetry, Srp Arh Celok Lek. 2010;138(5–6):297–299. 20607971 10.2298/sarh1006297j

[25] Masi A, Subcommittee for Scleroderma Criteria of the American Rheumatism Association Diagnostic and Therapeutic Criteria Committee. Preliminary criteria for the classification of systemic sclerosis (scleroderma). Arthritis Rheum 1980;23(5):581–590. 7378088 10.1002/art.1780230510

[26] Hoogen F, Khanna D, Fransen J, Johnson SR, Baron M. Tyndall A, et al 2013 classification criteria for systemic sclerosis: an American College of Rheumatology/European League Against Rheumatism collaborative initiative. Ann Rheum Dis 2013;65(11): 2737–2747. 10.1002/art.38098 PMC3930146 24122180

[27] Cutolo M, Sulli A, Smith V. Assessing microvascular changes in systemic sclerosis diagnosis and management. Nat Rev Rheumatol 2010;6:578–587. 10.1038/nrrheum.2010.104 20703220

[28] Horn A, Distler JH. Vascular alterations upon activation of TGFbeta signaling in fibroblasts—implications for systemic sclerosis. Arthritis Res Ther 2010;12(3):125 10.1186/ar3026 20602813 PMC2911883

