# Supplementary material for: Evaluation of Peripheral Blood Circulation Disorder in Scleroderma Patients Using an Optical Sensor with a Pressurization Mechanism
Source: PLoS One. 2016 Aug 1;11(8):e0159611. doi: 10.1371/journal.pone.0159611 (PMC4968816; doi:10.1371/journal.pone.0159611)
Supplement: S1 Dataset — P2 are 88, 64, 53, 40, and 28mmHg. (PDF) [file pone.0159611.s001.pdf]

ΔCHb [%mmm]						
	Age	P2				
		88mmHg	64mmHg	53mmHg	40mmHg	28mmHg
Healthy1	7?	0.03086	0.086687	0.140157	0.229077	0.354843
Healthy2	7?	0.021103	0.047117	0.08335	0.18561	0.417747
Healthy3	7?	0.044643	0.100893	0.12288	0.161347	0.195697
Healthy4	7?	0.065778	0.083015	0.108533	0.135423	0.14953
Healthy6	7?	0.03378	0.099868	0.17104	0.287555	0.457548
Healthy7	7?	0.008283	0.023917	0.033897	0.091957	0.159433
Healthy8	7?	0.01366	0.030967	0.059233	0.109097	0.198787
Healthy9	6?	0.01144	0.029427	0.070627	0.135923	0.297937
Healthy10	7?	0.03589	0.121927	0.173237	0.22245	0.274797
Healthy11	7?	0.011143	0.081433	0.13776	0.22457	0.339443
Healthy12	7?	0.037047	0.057877	0.117703	0.156003	0.235133
Healthy13	6?	0.151203	0.220597	0.307297	0.35351	0.36362
Healthy14	7?	0.012155	0.02353	0.03829	0.073475	0.168075
Healthy15	7?	0.01068	0.018583	0.026627	0.039703	0.141057
Healthy16	6?	0.03228	0.102975	0.109895	0.12218	0.19811
Healthy17	7?	0.046557	0.141333	0.204713	0.312107	0.390817
Healthy18	7?	0.04746	0.13391	0.180715	0.253045	0.31983
Healthy19	7?	0.0173	0.037317	0.065217	0.07436	0.1265
Healthy20	7?	0.01894	0.03247	0.03375	0.06378	0.093667
Healthy21	6?	0.034853	0.115447	0.166213	0.261873	0.362427
Healthy22	6?	0.015777	0.038743	0.050453	0.065023	0.108717
Healthy23	7?	0.121267	0.252867	0.34062	0.44494	0.623763
Healthy24	7?	0.011947	0.024917	0.031783	0.049983	0.08403
Healthy25	7?	0.0149	0.034953	0.08092	0.191443	0.360943
Healthy26	7?	0.013437	0.03889	0.057537	0.09109	0.150907
Scleroderma1	6?	0.012477	0.01514	0.02082	0.02703	0.041503
Scleroderma2	5?	0.014767	0.022053	0.018183	0.035013	0.058333
Scleroderma3	2?	0.016113	0.027493	0.027833	0.034273	0.047733
Scleroderma4	4?	0.011287	0.02741	0.037693	0.06031	0.09696
Scleroderma5	4?	0.03084	0.04842	0.06133	0.07587	0.08208
Scleroderma6	6?	0.026967	0.050727	0.08297	0.11508	0.176023
Scleroderma7	6?	0.016075	0.01553	0.01613	0.01769	0.024125
Scleroderma8	6?	0.02028	0.032	0.039397	0.041657	0.039713
Scleroderma9	6?	0.00693	0.022755	0.037375	0.07291	0.12579
Scleroderma10	5?	0.008773	0.00568	0.01057	0.018157	0.037213
Scleroderma11	7?	0.012523	0.06679	0.113927	0.172523	0.35129
Scleroderma12	6?	0.007143	0.013103	0.02158	0.04307	0.098817
Scleroderma13	7?	0.00842	0.012553	0.0234	0.036463	0.068817
Scleroderma14	7?	0.023137	0.014243	0.01454	0.022873	0.167653
Scleroderma15	6?	0.02199	0.05228	0.088717	0.161557	0.297003
Scleroderma16	6?	0.00948	0.009433	0.01188	0.010487	0.01627
Scleroderma17	6?	0.020063	0.02477	0.03075	0.043613	0.066813
Scleroderma18	6?	0.026	0.054627	0.096633	0.160683	0.293697
Scleroderma19	6?	0.022817	0.04833	0.074817	0.141783	0.26335
Scleroderma20	4?	0.007467	0.009227	0.013673	0.023387	0.034023
Scleroderma21	7?	0.005757	0.011673	0.020957	0.02719	0.081347
Scleroderma22	3?	0.011013	0.009803	0.008873	0.011137	0.013367
Scleroderma23	6?	0.01219	0.01015	0.010097	0.00954	0.012933
Scleroderma24	6?	0.01282	0.02435	0.03428	0.047023	0.10533
Scleroderma25	6?	0.018627	0.023487	0.030993	0.040937	0.049367
Scleroderma26	3?	0.006027	0.021317	0.03237	0.07487	0.14206
